# Un tatouage bulleux

**DOI:** 10.11604/pamj.2015.20.147.3592

**Published:** 2015-02-17

**Authors:** Najwa Guerouaz, Badredine Hassam

**Affiliations:** 1 Service de Dermatologie, Vénérologie, CHU Ibn Sina, Rabat, Maroc

**Keywords:** Tatouage, paraphénylène diamine, lésions bulleuses, Tattoo, p-phenylenediamine, bullous lesions

## Image en medicine

La paraphénylène diamine (PPD) est un colorant industriel connu dans le milieu médical par sa haute toxicité systémique au cours des tentatives d'autolyse. Son utilisation dans le domaine cosmétique connait un essor considérable dans les pays en voie de développement. O.M, âgé de 19 ans, est suivi pour rhinite allergique. Il a consulté pour des lésions bulleuses prurigineuses des deux avant-bras apparues 10 jours après un tatouage par une teinte à cheveux. L'examen clinique notait des lésions papulovésiculeuses uniformes reprenant exactement le graffiti initialement dessiné. L'enquête toxicologique centrée sur le produit du tatouage a précisé que ce dernier était à base de PPD. Le diagnostic d'eczéma de contact à la PPD fut retenu et une notification au centre antipoison national fut transmise. Le traitement par dermocorticoïdes de classe III était efficace avec amendement des lésions cutanées au bout de 10 jours. Dans les tatouages éphémères, la PPD est utilisée seule ou additionné au henné vert naturel pour augmenter la longévité du motif. Cependant, ce produit peut être à l'origine de réactions allergiques sévères survenant des heures, voire des semaines, après la réalisation du tatouage. La réaction la plus fréquente est la dermatite de contact où la sensibilisation est définitive et irréversible d'où l'obligation d'une éviction définitive.

**Figure 1:**
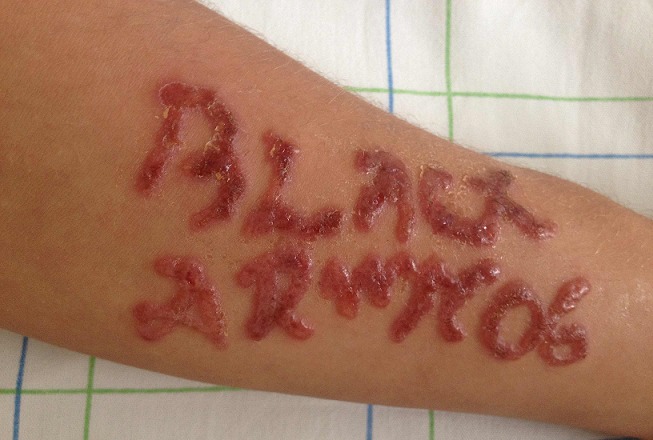
Lésions papulovésiculeuses reprenant un motif graphique

